# Genome-wide recombination map construction from single sperm sequencing in cattle

**DOI:** 10.1186/s12864-022-08415-w

**Published:** 2022-03-05

**Authors:** Liu Yang, Yahui Gao, Mingxun Li, Ki-Eun Park, Shuli Liu, Xiaolong Kang, Mei Liu, Adam Oswalt, Lingzhao Fang, Bhanu P. Telugu, Charles G. Sattler, Cong-jun Li, John B. Cole, Eyal Seroussi, Lingyang Xu, Lv Yang, Yang Zhou, Li Li, Hongping Zhang, Benjamin D. Rosen, Curtis P. Van Tassell, Li Ma, George E. Liu

**Affiliations:** 1 Animal Genomics and Improvement Laboratory, Henry A. Wallace Beltsville Agricultural Research Center, Agricultural Research Service, USDA, Beltsville, MD 20705 USA; 2 College of Animal Science and Technology, Sichuan Agricultural University, Chengdu, 611130 China; 3 Department of Animal and Avian Sciences, University of Maryland, College Park, MD 20742 USA; 4 College of Animal Science and Technology, Yangzhou University, Yangzhou, 225009 China; 5 College of Animal Science and Technology, China Agricultural University, Beijing, 100193 China; 6 College of Agriculture, Ningxia University, Yinchuan, 750021 China; 7 Animal Nutritional Genome and Germplasm Innovation Research Center, College of Animal Science and Technology, Hunan Agricultural University, Changsha, 410128 China; 8 Select Sires Inc, 11740 U.S. 42 North, Plain City, OH 43064 USA; 9 The Roslin Institute, Royal (Dick) School of Veterinary Studies, The University of Edinburgh, Midlothian, EH25 9RG UK; 10 Division of Animal Sciences, University of Missouri, Columbia, MO 65201 USA; 11 Agricultural Research Organization (ARO), Volcani Center, Institute of Animal Science, P.O.B 15159, HaMaccabim Road, 7528809 Rishon LeTsiyon, Israel; 12 Innovation Team of Cattle Genetic Breeding, Institute of Animal Sciences, Chinese Academy of Agricultural Sciences, Beijing, 100193 China; 13 Key Laboratory of Agricultural Animal Genetics, Breeding and Reproduction of Ministry of Education & College of Animal Science and Technology, Huazhong Agricultural University, Wuhan, 430070 China

**Keywords:** Cattle, Single sperm, Sequencing, Recombination

## Abstract

**Background:**

Meiotic recombination is one of the important phenomena contributing to gamete genome diversity. However, except for human and a few model organisms, it is not well studied in livestock, including cattle.

**Results:**

To investigate their distributions in the cattle sperm genome, we sequenced 143 single sperms from two Holstein bulls. We mapped meiotic recombination events at high resolution based on phased heterozygous single nucleotide polymorphism (SNP). In the absence of evolutionary selection pressure in fertilization and survival, recombination events in sperm are enriched near distal chromosomal ends, revealing that such a pattern is intrinsic to the molecular mechanism of meiosis. Furthermore, we further validated these findings in single sperms with results derived from sequencing its family trio of diploid genomes and our previous studies of recombination in cattle.

**Conclusions:**

To our knowledge, this is the first large-scale single sperm whole-genome sequencing effort in livestock, which provided useful information for future studies of recombination, genome instability, and male infertility.

**Supplementary Information:**

The online version contains supplementary material available at 10.1186/s12864-022-08415-w.

## Background

Meiotic recombination promotes genetic diversity by reshuffling parental alleles and providing novel combinations of genes for evolutionary selection [1–5]. Recombination is also crucial for ensuring proper segregation of homologous chromosomes during meiosis [4]. Considerable variations in recombination rates between individuals have been documented in human and other species [6–10].

Recombination hotspots are usually clustered into narrow genomic regions specified by the PR domain-containing 9 (*PRDM9*) gene in human and mouse [11–15]. *PRDM9* has driven evolutionary erosion of hotspots in *Mus musculus* through haplotype-specific initiation of meiotic recombination [16]. Since crossovers were disfavored at such hotspots, sequence divergence generated by hotspot turnover may create an impediment for recombination in hybrids, potentially leading to reduced fertility and thus, eventually, speciation [17, 18]. More recent publications investigated the rules governing DNA recombination, revealing the relationships between the distribution of crossovers, proteins involved in recombination, and specific factors determining whether a double-strand break becomes a crossover [19, 20].

Besides popular pedigree-based studies, there exist two other methods for measuring recombination based on sperm typing or linkage disequilibrium (LD) patterns. Single-sperm genomics and sperm typing can assess recombination in a regional or genome-wide [21, 22]. Using a single sperm isolation and sequencing approach, the Quake lab reported an average of 22.8 recombination events, 5 to 15 gene conversion events, as well as 25 to 36 de novo mutations in each human sperm [22]. Similarly, the Xie group reported aneuploidy in 4% of the cells and 26 recombination events per human sperm [23]. The Donnelly team later developed a method to sequence individual mouse sperm and applied it to mice carrying two different alleles of *PRDM9* in mammalian crossovers [20]. A new method called ReMIX was introduced to detect crossovers from gamete DNA using Illumina sequencing of 10X Genomics linked-read libraries in a single mouse and stickleback fish [24]. As a variation of Drop-seq [25], Sperm-seq is another high-throughput and low-cost approach to quantify recombination variation across the gamete genomes. Using Sperm-seq, Bell et al. sequenced 31,228 human sperm genomes from 20 men, identifying 813,122 crossovers and other genomic anomalies [26]. They discovered that crossover frequency and location, as well as other meiotic phenotypes like chromosome aneuploidy, vary across chromosomes, gametes, and human donors. The authors propose that inter-cell and inter-individual variation in meiotic chromosome compaction could partially explain this covariance.

Using large-scale cattle pedigree data, we have previously reported different recombination patterns between bulls and cows and identified several loci associated with recombination rate and hotspot usage in both sexes, including the *PRDM9* gene on chromosome 1 [27]. Similar results were also reported by other groups [28, 29]. In our second cattle study using single sperm genomics, we examined the allele pattern of *PRDM9* impacting cattle genome recombination [30]. Later, we also detected *Bos taurus*–*indicus* hybridization correlates with intralocus sexual-conflict effects of *PRDM9* on male and female fertility in Holstein cattle [31]. Here, we analyze 143 single sperm genomes from two Holstein bulls to derive two individualized recombination maps, identifying 4,291 crossovers. We further validated the reliability of single-sperm sequencing-based results, using the data derived from the diploid genome sequencing of one sample’s family trio and our previous recombination studies. To our knowledge, this is the first large-scale single sperm whole-genome sequencing report in livestock, which could facilitate future studies of recombination, genome instability, and male infertility.

## Results

### Sequencing and genotyping of haploid sperms and diploid trio

#### Sequencing for sperms

We chose two bulls with different fertility capabilities (See Methods). Using the MALBAC method [30], we successfully picked, amplified, and sequenced a total of 156 single sperm cells from two Holstein bulls’ semen. After quality control filtering, we kept 143 sperm data (71 for Sample1 and 72 for Sample2) for downstream analyses. The sequenced sperms had an average genome coverage depth of 1.79 × , and 16 of them had genome coverage depth of ~ 4 × , corresponding to an overall genome coverage of ~ 11.40% to ~ 41.35%, respectively (Table S1). On average, we mapped 98.18% of sequencing reads from single sperms on the bovine ARS-UCD1.2 genome.

#### Genotyping for sperms

We used GATK to call the raw genotypes for SNPs and INDELs [32]. Each sperm generated raw calls for 15.5—43.0 million SNPs and 2.4—7.2 million INDELs (Table S2). Since sperms are haploid cells, we removed extensive heterozygous genotype calls. Only a small fraction of heterozygous raw calls was detected, with an average frequency of 2.46% for SNPs (ranging from 1.03% to 7.39%) and 2.97% for INDELs (ranging from 1.03% to 9.16%), respectively. These data indicated that most of the sperms were isolated successfully with low contamination before sequencing. After strict filtration, we kept approximately 4.29% SNPs (ranging from 0.42 to 2.68 million) and 11.21% INDELs (ranging from 0.23 to 1.04 million). Compared to our previous single sperm recombination analysis using the BovineHD SNP chip [30], our current study covered ~ 20 fold more clean SNPs, with an average of 1.12 million (Table S2).

#### Trio

For Samples1’s family trio diploid genomes, we sequenced bulk DNA samples extracted from ear punches of Sample1, its sire Sample1-sire, and dam Sample1-dam to approximately 40 × , 10 × , and 20 × genome coverage, respectively, with over 99% genome mapping rate and covering 96% genome sequence (Table S3). After QC filtering, we obtained approximately 5.61 million (62.89%) SNPs and 0.72 million (65.26%) INDELs of Sample1. Within them, 44.45% and 46.48% high-quality SNPs and INDELs were heterozygous, respectively (Table S4).

### Individual recombination maps

#### Phasing

As described in Methods, assuming the low probability of crossovers between nearby SNPs, we phased the heterozygous genotypes of the bulls into haplotypes based on sperm linkage information. In 71 Sample1 sperms and 72 Sample2 sperms, a total of 310,271 and 307,451 autosomal heterozygous SNPs (htSNPs) were phased, and the phasing rates were 85.79% and 80.40%, respectively (Table 1, Table S5, and Table S6). To verify the phased haplotypes, we phased a total of 1,501,331 (79.81%) htSNPs from Sample1 using its family trio information. We used that as a scale plate to estimate the agreement rate of phased sperm alleles. Totally, 173,157 htSNPs for Sample1 were phased by either single sperm haploid genomes or Sample1 trio diploid genomes, and 95.22% (164,885) of them were consistent between alleles phased by both.

**Table 1:** Statistics of recombination events in sperms

Sperms	Covered htSNP	Phased SNP	Phased rate	Crossover	Morgan	SD	SE	R rate (cM/Mb)
Total	744,063	617,722	83.02%	4291	30.01	9.12	0.76	1.21
Sample1	361,651	310,271	85.79%	2012	28.34	9.46	1.12	1.14
Sample2	382,412	307,451	80.40%	2279	31.65	8.52	1.00	1.27

#### Crossover

With the phased autosomal htSNPs of Sample1 and Sample2, we inferred their crossovers occurred in the interval region of htSNPs using an HMM method, as previously described [30]. The 143 single sperms gave a total of 4,291 crossover events, on average ~ 30.01 ± 0.76 standard error (SE) (9.12 SD) per sperm (Table S7). An average of ~ 32 Mb distance between two crossovers was observed on those chromosomes with double crossovers (Fig. S1). Approximately 80.3%, 64.6%, and 37.0% of the total crossovers can be confidently localized to intervals of 200, 100, and 30 kb, respectively (Fig. S2). The resolutions of our cattle recombination results were between the outcomes from two previous human studies, where their corresponding percentages were: 59%, 37%, and 13% [22] as well as 93%, 80%, and 45% [23] at those three interval thresholds, respectively.

**Fig. 1:**
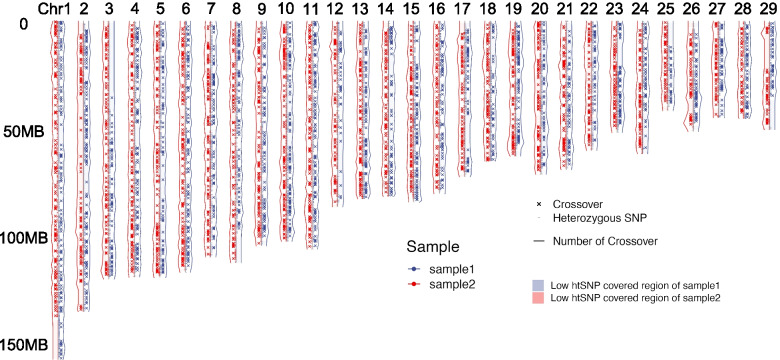
Genome-wide distribution of recombination crossovers for two Holstein bulls. Sample1: red and Sample2: blue. The crossover position is denoted in the center of two htSNP intervals. Solid lines represent the frequencies of crossover in 1 Mb window size. The low htSNP density regions (gray regions) were inferred by the HMM method as regions with htSNP less than 50 per Mb

**Fig. 2:**
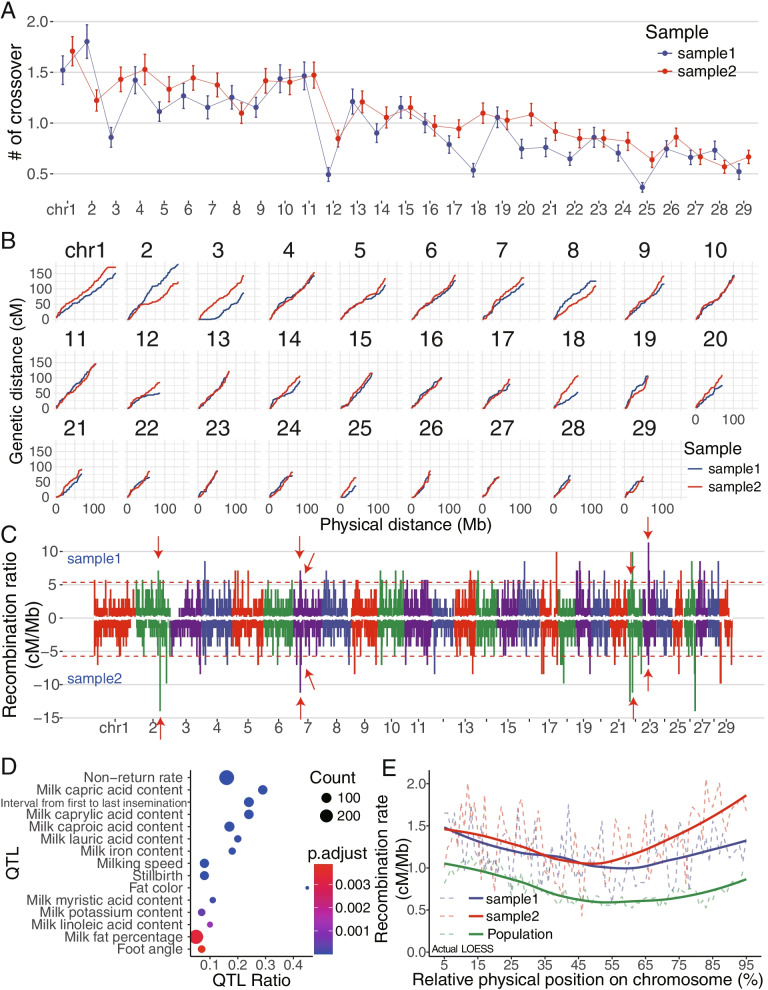
Individual recombination maps. **A** The average number of crossovers for two samples in each chromosome. **B** Recombination maps of two samples. Accumulated relationships of the physical and genetic length of each chromosome. **C** Recombination rate per Mb in each chromosome. Red dotted lines represent thresholds of 2.5 standard deviations away from the mean genome-wide recombination rate. Chromosomes were represented in different colors. Five shared common hot spots were labeled by arrows. **D** QTL enrichment of recombination hotspots. Significance was determined by Fisher's exact test, and *p-*values were adjusted for multiple comparisons by the Benjamini and Hochberg's (BH) algorithm. **E** Distribution of the autosomal recombination rates over chromosomes. The curves are smoothed by the LOESS method

When comparing the two Holstein bulls Sample1 and Sample2, we constructed individual recombination maps for all chromosomes, spanning 28.34 ± 1.12 SE (9.46 SD) Morgans in Sample1 and 31.65 ± 1.00 SE (8.52 SD) Morgans in Sample2, respectively (Fig. 1 and Table S8). Fewer crossovers were identified in some low htSNP density regions, for example, in runs of the homozygous region (ROH) in BTA 2, 3, 12, and 18 of Sample1 when compared to Sample2. The low htSNP density regions also had large distances between htSNPs. When testing the relationship between the numbers of crossovers and the chromosome length, we did not find a strong correlation within these low htSNP density regions (ANOVA type III, *P*-values = 0.076). To control the ROH effects, we removed 75 regions covered by less than 50 htSNP per Mb of the genome for the two donors in all subsequent analyses (Fig. S3 and Table S9). As shown in Fig. 2A, after removing the low htSNP density regions, the number of crossovers on chromosomes increased with the chromosome length (Fig. S4). Besides, the individual recombination maps of Sample1 and Sample2 showed that most of the chromosomes are broadly similar, with differences found in chr2, chr3, and chr28 (Fig. 2B).

#### Hotspot

The recombination crossover locations were not uniformly distributed along the genome in these two individual bulls. We defined recombination hotspot as a short chromosomal region where crossovers occur more frequently than in other regions, as described previously [27]. In brief, we defined the recombination hotspots in these two individuals as the regions with a recombination rate of 2.5 × SD greater than the mean. We detected a total of 103 (4.14% of total autosomes) hotspots in Sample1, and 41 (1.65%) hotspots in Sample2 (Table S10), with five of them, shared between the two samples (Fig. 2C). When overlapping with the bovine quantitative trait loci (QTL) database [33], these 139 hotspots were significantly enriched in 31 bovine QTL, such as non-return rate, the interval from first to last insemination, and milk-composition-related QTL (Fig. 2D and Table S11).

#### Compare sperm recombination maps to our earlier cattle recombination results

We also checked the consistency of recombination patterns derived from individual sperm sequencing compared to those from pedigree data [27] and individual sperm genotyping by the Illumina BovineHD BeadChip [30]. Because the pedigree data were based on SNP chips, the recombination events were usually underestimated within the first and last 5 Mb distal, i.e., terminal regions of chromosomes. After excluding these regions, we converted the recombination intervals to a Mb scale, assuming 1 centimorgan or cM corresponding to 1 Mb.

Notably, the crossover hotspots were enriched in both ends of chromosomes, which corresponds to chromosomal pericentromeric and subtelomeric regions, as all bovine autosomes are acrocentric. For the same individual (Sample1), its sperm recombination maps based on sequencing or BovineHD SNP array genotyping showed a similar pattern and level, except for in the proximal regions, where sperm sequencing showed a trend of higher recombination rates (Fig. 2E). When we compared the individual sperm sequencing recombination maps (Sample1 and Sample2) to the pedigree-based population recombination map, we also detected a similar pattern (Pearson correlation coefficient of the curves between Sample1 and Sample2, Sample1 and population, and Sample2 and population are 0.677, 0.946, and 0.494 respectively, all *P*-values < 2.2e-16). But we also found that the recombination rates from single sperm sequencing were generally higher than those reported from population pedigree-based data (Fig. 2E).

## Discussion

Although meiotic recombination is known to enhance genetic and phenotypic variations, it is also variable and error-prone: recombination rates vary among sperms, chromosomes, and individuals. Chromosome missegregation can cause abnormal chromosome numbers (aneuploidy), while non-allelic homologous recombination leads to over two-thirds of the structural variation detected within the human genome [34]. The purpose of this study was to probe meiotic recombination in cattle sperm.

The resolution of our cattle recombination maps is close to the previous human study [23]. The minor variances could be partially due to differences in species, platforms of whole genome amplification, quality control, and/or other factors. Given that sampling and genotype errors may potentially bias the pedigree-based results, we further confirmed our findings using the family trio diploid genome sequencing and our previous recombination study based on cattle pedigree. Our average sequencing depth is ~ 1.79 × , and genome coverage is from ~ 11.40% to ~ 41.35% per sperm, which are equivalent to the human study with the corresponding numbers of ~ 1 × depth and 11–44% genome coverage [23]. The Sperm-seq numbers are even lower, with 0.02 × depth and 1% genome coverage [26]. Since these are typical for single sperm assays, in silico simulations or comparisons with known haplotypes were often used to verify the phasing results [23] [26]. We also sequenced the genomes from the donor’s parents and used a pedigree approach to infer the phase information of the donor. We obtained 95.22% consistency, indicating the high accuracy of our approach in phasing htSNPs into chromosome-level haplotypes. In addition, the individual recombination maps of Sample1 and Sample2 showed that most of the chromosomes are broadly similar, with differences found in chr2, chr3, and chr28 (Fig. 2B). These differences also agree with previous publications, which reported that some recombination hotspots are evolving and individual-specific [35]. Interestingly, there are differences in terms of fertility traits for Sample1 and Sample2 (See Methods).

Although the genome-wide recombination distributions from these two approaches were consistent, we found the recombination rates from single sperm sequencing are generally higher than those from population pedigree-based data (Fig. 2E). These findings generally agreed with the earlier human results [22], which showed that the recombination maps from the pedigree and sperm-typing methods were largely consistent, but considerable differences were detected at a higher resolution. Because the sperms used in our study were active and viable, the differences in fitness were small between them. Therefore, different recombination patterns between sperms and live-born offspring could be caused by the selection processes during egg-sperm fertilization and embryo development till birth. Although it is intuitively unclear what factors drive such differences, based on our results and previous reports [33], we postulate the selection process between sperm-egg fertilization and embryo development to be plausible explanations. We found that a trend of higher recombination rates in the proximal regions was detected by single sperm sequencing than by the BovineHD SNP array genotyping of the same bull sperms. We partially attributed it to that sequencing could report more htSNPs than the SNP array. One limitation is that only two Holstein bulls were used in this study, so it is hard to obtain the recombination patterns within a population. The recently reported Sperm-seq will make it possible to survey more sperms in large number of samples more efficiently [26].

In conclusion, using single sperm sequencing, we investigated occurrences and distribution patterns of meiotic recombination in cattle sperm. Our results mainly agree with previous outcomes derived from population pedigree-based data, sperm typing, and family trio diploid sequencing experiments. To our knowledge, this is the first large-scale single sperm cell sequencing report in livestock, which will further enable future studies of sperm genome instability and male infertility.

## Methods

### Sample collection and whole genome amplification and sequencing

We chose two Holstein bulls with different fertility capabilities: Sample1 has a DPR (daughter pregnancy rate) PTA (Predicted Transmitting Ability) value of 0.0, reliability of 0.99, estimated from 6,528 daughters. In contrast, Sample2 has a DPR PTA value of -3.2, reliability of 0.99, estimated from 15,314 daughters. Their pedigree relationship is 0.127 and the genomic relationship is 0.08, which are close to the relationship of cousins. Both are heterozygous for PRMD9 locus (allele 5/non allele 5). They were chosen based on their contrasting daughter pregnancy rates. Somatic tissue (ear punch) samples of Holstein Sample1, together with its parent somatic tissues, were donated by Select Sires, Inc (Plain City, OH, USA). Semen samples were freshly collected by Select Sires, Inc. in its routine artificial insemination semen straw production. After receiving them under liquid nitrogen in USDA-ARS Animal Genomics and Improvement Laboratory (AGIL), we manually isolated a total of 156 sperm cells from two Holstein bulls (Sample1 with 73 sperm cells and Sample2 with 83 sperm cells). Briefly, isolated sperms were thawed in 37 ℃ water for 30-45 s and treated with 0.25% Trypsin–EDTA, followed by dilution with PBS + 1% BSA and washing twice. The sperms were further diluted to a proper resolution using PBS + 1% BSA on a petri-dish. Active single sperms were picked up manually by pipetting into a reaction tube under a micromanipulator described previously [30]. Whole-genome amplification was performed on single cells according to the manufacturer’s protocol, using the Single Cell Whole Genome Amplification Kit developed from the Multiple Annealing and Looping Based Amplification Cycles (MALBAC, Yikon Genomics, Shanghai, China) method [36]. In brief, a single sperm was initially analyzed and pre-amplified by primers supplied in the kit with 8 cycles with multiple annealing steps. PCR generated fragments with variable lengths at random starting positions for next-generation sequencing. To evaluate the agreement rate of individual recombination from sperms and parents, we also sequenced the somatic diploid genomes of the trio, including Sample1 (Sample1-diploid) and its parents (Sample1-sire and Sample1-dam). Using their somatic ear punch tissues, we isolated their diploid genomes using a QIAGEN QIAamp DNA Mini Kit protocol (QIAGEN, Valencia, CA, USA). DNA extracted from the ear skin samples of the donor and his parents was then used for preparing sequencing libraries using standard Illumina TruSeq Library Prep Kit and sequenced on an Illumina HiSeq 2000/NextSeq 500 sequencing platform with read length of PE150 (Illumina, San Diego, CA).

### Genotype calling

Paired-end sequencing reads for single sperm, and diploid samples were quality controlled by fastqc v0.11.9 and trimmed by Trimmomatic v0.39 [32]. Bwa v0.7.17 mem was used with default parameters to align clean reads against the bovine reference genome ARS-UCD1.2 (ftp://ftp.ensembl.org/pub/release-99/fasta/bos_taurus/dna/Bos_taurus.ARS-UCD1.2.dna.toplevel.fa.gz). To avoid potential PCR or sequencing optical artifacts, we marked duplicated reads that were mapped to the same location by MarkDuplicates function in GATK v4.0.8.1 [32]. FixMateInformation was also employed to ensure all mate-pair information is in sync between each read and its mate-pair. For detecting systematic errors made by the sequencing machine, Base Quality Score Recalibration (BQSR) was called for each BAM by BaseRecalibrator and ApplyBQSR with the known single nucleotide polymorphism (SNP) file from 1000 Bull Genomes Projects (http://www.1000bullgenomes.com/) [32]. HaplotypeCaller in GATK was used to call variants, and the parameter -ERC GVCF in CombineGVCFs was set for data combining and then performed by GenotypeGVCFs [32]. We separated SNPs and INDELs (short insertion and deletion) in a combined VCF file using the function SelectVariants, respectively.

### Filtration of SNPs, INDELs, and samples

To improve the genotyping accuracy for single sperms, we applied a stringent cutoff on the raw genotyping quality score to call genotypes [32]. We removed low-quality variants with quality by depth (QD) < 2, Fisher strand (FS) > 30, strand odds ratio (SQR) > 3, root mean square of the mapping quality (MQ) < 40, and quality score (QUAL) < 40. Using the VariantFiltration function in GATK, we defined the window size as 35 to evaluate clustered SNPs and allowed three SNPs to make up a cluster. For sperm data, we kept variants with at least 2 allele support reads and removed heterozygous (0/1) SNPs or INDELs because it was potentially caused by sequencing error or sperm chromosome-scale genomic anomalies [26]. As a result, 12 sperm samples were removed as their read depth was lower than 0.5X (10 sperms) or genome coverage rate lower than 10% (2 sperms). In addition, for diploid data, we filtered those variants with allele support reads less than 1/2 genome-wide depth [32].

### Inferring haplotype with sperm

We used two different genotypes—reference allele (0) and first alternate allele (1) in sperms to infer haplotypes. To avoid large numbers of unbalances between these two alleles, we only kept those sites with the minimum frequency of 30% for either allele with at least two supporting sperms. Based on sperm linkage information, we inferred haplotypes using the previously published two-stage method [23], with some modifications for our strict filtration parameters. First, we constructed a haplotype profile using a fraction (10%) of htSNPs covered by more than 20 sperm SNPs. Based on genome coordinates, we linked every two neighboring htSNPs and generated four potential combinations. As the rates of false SNP calling and recombination are low, the true links will appear much more frequently than the false links based on the frequency of neighboring htSNP pairs in all sperm data. We defined two true links that appeared eight times and two false links that occurred no more than once for a neighboring htSNP pair. If data were not satisfying these criteria, the first htSNPs would be linked to the next htSNPs until the true links appear eight times. The htSNPs satisfying these criteria were phased into one of the two haplotypes. We then imputed missing htSNPs into the haplotypes. In each sliding window of five phased htSNPs sorted by genome coordinate, those missing htSNPs were imputed recursively into either haplotype if one sperm cell had at least three confirmed phased htSNPs. To improve the phasing rate, we further imputed the remaining genotypes by borrowing information across sperm cells. We selected the top 10 sperms sorted by the genotype concordance rate with either phased haplotype. The sperm with missing htSNPs were imputed into a haplotype if two or more sperms covered this haplotype, and this haplotype had a larger number of sperm cell counts than the other haplotype. This imputation was performed for both haplotypes. After these two stages, over 80% of the htSNPs were phased into chromosome-level haplotypes for both bulls.

### Phasing haplotype by Sample1 trio information

To estimate the agreement of phased haplotype of single sperms, we also sequenced the diploid genome of Sample1 and its parents. In genetics, diploid genotypes include one paternal allele and one from maternal in normal conditions, and the mutation rate is very low. Based on SNP linkage information, we phased the heterozygous genotype of Sample1 to paternal haplotype and maternal haplotype. For example, assuming the heterozygous genotype of offspring is ‘AG’. Three conditions can phase ‘A’ into paternal haplotype and ‘G’ into maternal haplotype: the father’s genotype is ‘AA’ and mother’s genotype is ‘GG’ at this SNP; the father’s is ‘AG’ and mother’s is ‘GG’; or the father’s is ‘AA’ and mother’s is ‘AG’.

### Inferring crossover in single sperms

The Viterbi algorithm in a Hidden Markov Model (HMM) were applied to infer the most likely states of sequence along the genome based on phased htSNPs of single sperms [20]. A crossover event occurred in the transition of a window between two htSNPs. For each chromosome of sperms, we randomly transformed a haplotype as paternal and the other one as maternal. One sample with abnormal numbers of crossovers was excluded. To avoid the genetic background, such as runs of the homozygous region (ROH) influencing the comparison of individual recombination patterns, we applied the HMM method for excluding the low htSNP density region with htSNP less than 50 per Mb across sperms of two samples.

## Supplementary Information

**Additional file 1. **

**Additional file 2. **

## Data Availability

The data that support the results of this research are available within the article and its Supplementary Information files. All other sequence data can be tracked in supplemental files. The single sperm sequencing data were submitted to GEO under the accession number PRJNA691741 (https://dataview.ncbi.nlm.nih.gov/object/PRJNA691741?reviewer=kj8n0f06eekt1uck7726jijms3).

## Abbreviations

AGIL: Animal Genomics and Improvement Laboratory
DPR: Daughter pregnancy rate
GWAS: Genome-wide association study
HMM: Hidden Markov model
htSNP: Heterozygous SNP
INDEL: Short insertion and deletion
kb: Kilobase pairs
LD: Linkage disequilibrium
MALBAC: Multiple annealing and looping based amplification cycles
Mb: Megabase pairs
PRDM9: PR domain-containing 9
QC: Quality control
QTL: Quantitative trait loci
SD: Standard deviation
SE: Standard error
SNP: Single nucleotide polymorphism
