# Supplementary material for: Genome-wide recombination map construction from single sperm sequencing in cattle
Source: BMC Genomics. 2022 Mar 5;23:181. doi: 10.1186/s12864-022-08415-w (PMC8898482; doi:10.1186/s12864-022-08415-w)
Supplement: Supplementary file 1 — Additional file 1. [file 12864_2022_8415_MOESM1_ESM.pdf]

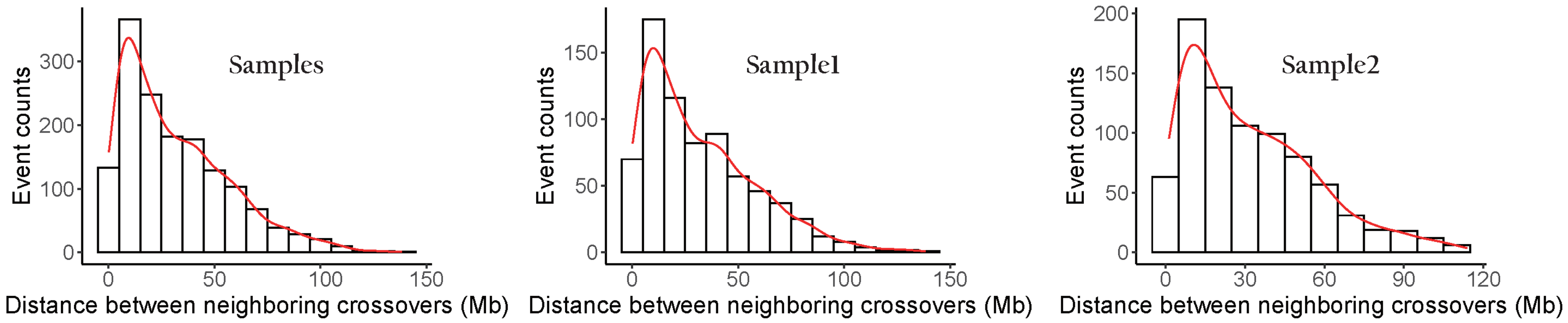

Figure S1. Histograms of crossover interference. Here the gamma distribution was fit for crossover pairs. Significant deviations from random distribution were observed, indicating crossover interference exists through chromosomes for all samples (left), Sample1 (middle), and Sasmple2 (right)

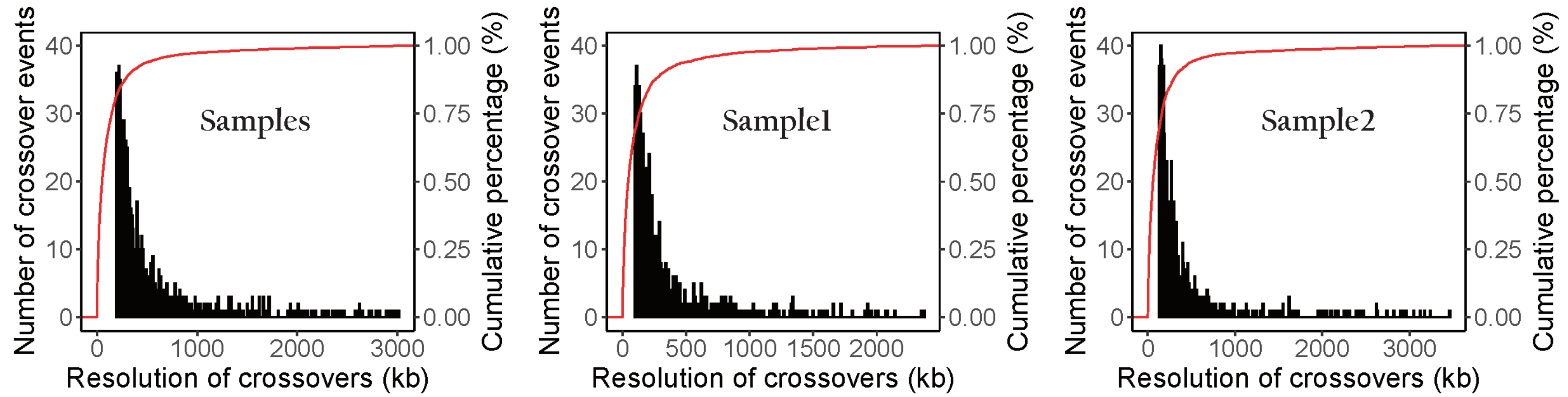

Figure S2. Histograms of crossover resolution for all samples (left), Sample1 (middle), and Sasmple2 (right). Approximately 80.3%, 64.6%, and 37.0% of the total crossovers can be confidently localized to intervals of 200, 100, and 30kb, respectively.

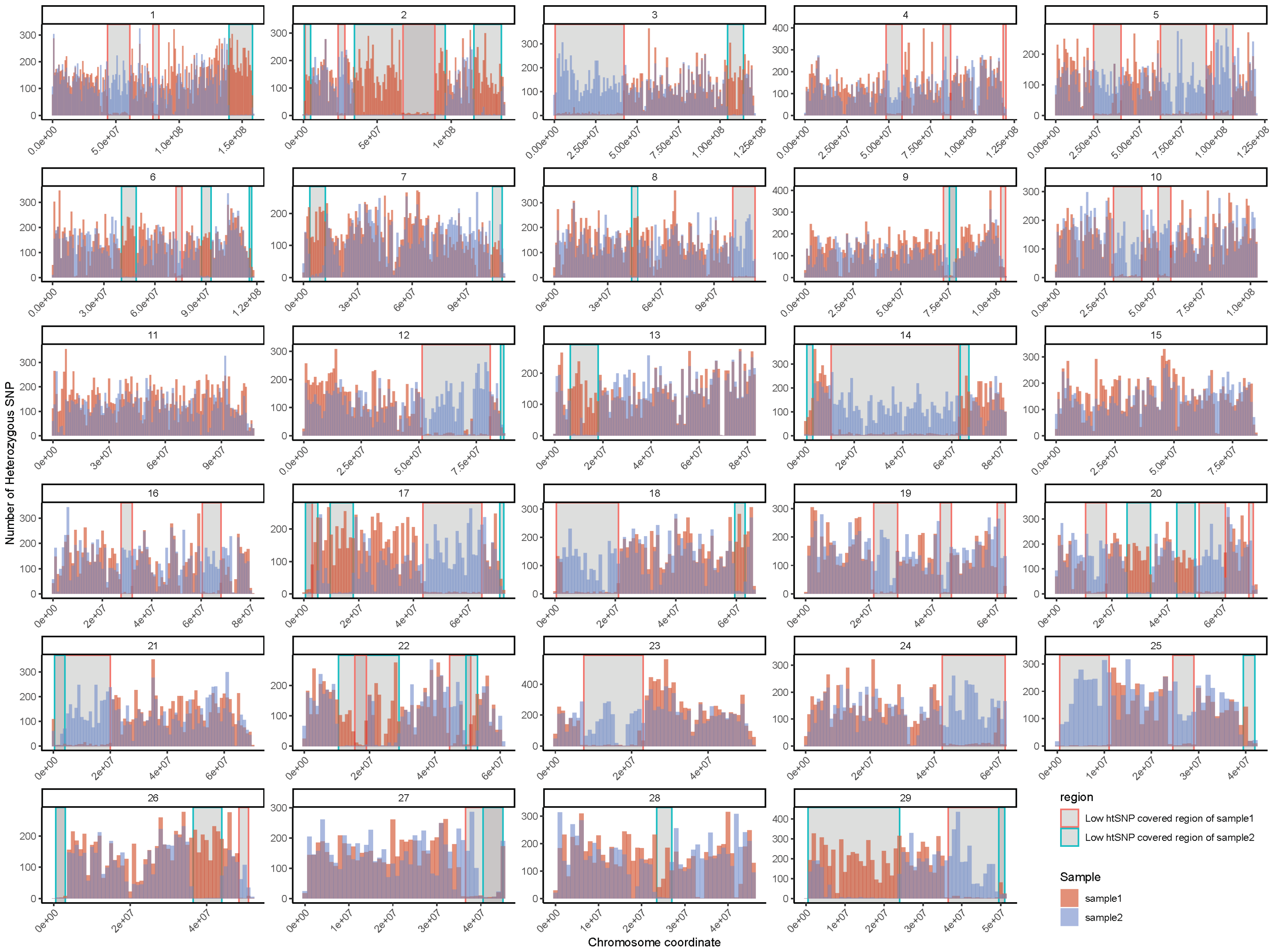

Figure S3. Seventy-five regions of low htSNP density across the cattle chromosomes in two bulls Sample 1 (red) and Sample2 (blue).

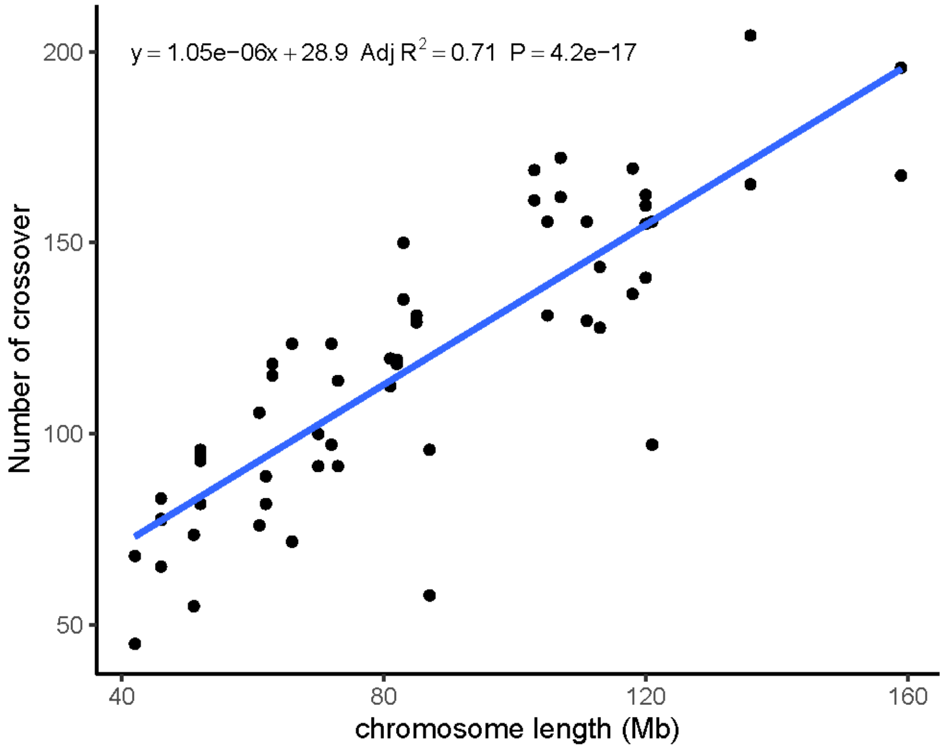

Figure S4. The number of crossovers on chromosomes increased with the chromosome length, after removing 75 low htSNP density regions.
